# Resonant Zener tunnelling via zero-dimensional states in a narrow gap diode

**DOI:** 10.1038/srep32039

**Published:** 2016-08-18

**Authors:** D. M. Di Paola, M. Kesaria, O. Makarovsky, A. Velichko, L. Eaves, N. Mori, A. Krier, A. Patanè

**Affiliations:** 1 School of Physics and Astronomy, The University of Nottingham, Nottingham NG7 2RD, UK; 2 Physics Department, Lancaster University, Lancaster LA1 4YB, UK; 3 Graduate School of Engineering, Osaka University, 2-1 Yamada-Oka, Suita City, Osaka 565-0871, Japan

## Abstract

Interband tunnelling of carriers through a forbidden energy gap, known as Zener tunnelling, is a phenomenon of fundamental and technological interest. Its experimental observation in the Esaki *p-n* semiconductor diode has led to the first demonstration and exploitation of quantum tunnelling in a condensed matter system. Here we demonstrate a new type of Zener tunnelling that involves the resonant transmission of electrons through zero-dimensional (0D) states. In our devices, a narrow quantum well of the mid-infrared (MIR) alloy In(AsN) is placed in the intrinsic (*i*) layer of a *p-i-n* diode. The incorporation of nitrogen in the quantum well creates 0D states that are localized on nanometer lengthscales. These levels provide intermediate states that act as “stepping stones” for electrons tunnelling across the diode and give rise to a negative differential resistance (NDR) that is weakly dependent on temperature. These electron transport properties have potential for the development of nanometre-scale non-linear components for electronics and MIR photonics.

Zener tunnelling^1^ has played a key role in the understanding of quantum phenomena in condensed matter physics. Since its experimental observation in the Esaki diode^2^, it has been investigated in several material systems. Renewed interest in Zener tunnelling has emerged from the research on graphene^3^, where relativistic electrons can tunnel through a potential barrier without back scattering, a phenomenon called Klein tunnelling^4^. On the other hand, Zener tunnelling via zero dimensional (0D) states remains largely unexplored due to lack of suitable structures and materials for its observation. Here we report on Zener tunnelling involving 0D states formed by the incorporation of N-atoms in the narrow gap semiconductor InAs.

The high mobility of electrons in InAs has enabled the discovery of new excitations of matter^5^, large magneto-resistance effects^6^, and high-performance electronic devices^7^. Also, the additional narrowing of the band gap of InAs by a small concentration of N-atoms (<3%) has offered new routes to tuneable photon absorption in the technological important mid-infrared (MIR) spectral gap (λ > 3 μm)^8^,^9^,^10^. The N-induced narrowing of the band gap arises from the hybridization of the extended conduction band (CB) states of InAs with the N-energy levels located above the CB edge^10^,^11^,^12^; a more complex picture can also arise from crystalline defects, such as N-clusters^13^ and/or point defects^14^,^15^, thus leading to a band structure with admixed localized and delocalized states. In this work, we demonstrate that the localized levels induced in the band gap by the N-incorporation provide intermediate states as “stepping stones” for tunnelling of electrons across the *p*-*n* junction of a resonant tunnelling diode (RTD), thus leading to a negative differential resistance (NDR) that is only weakly affected by temperature. We probe these localized states by magneto-tunnelling spectroscopy (MTS) and demonstrate that they are strongly confined at small length scales, λ_0_ ~ 1.5 nm, with binding energies much larger than for shallow donors in InAs. The N-induced localized states in InAs provide a novel means of tailoring diode characteristics for electronic and MIR photonic applications.

## Results

### Resonant tunnelling diodes based on the narrow gap In(AsN)

**Figure 1:**
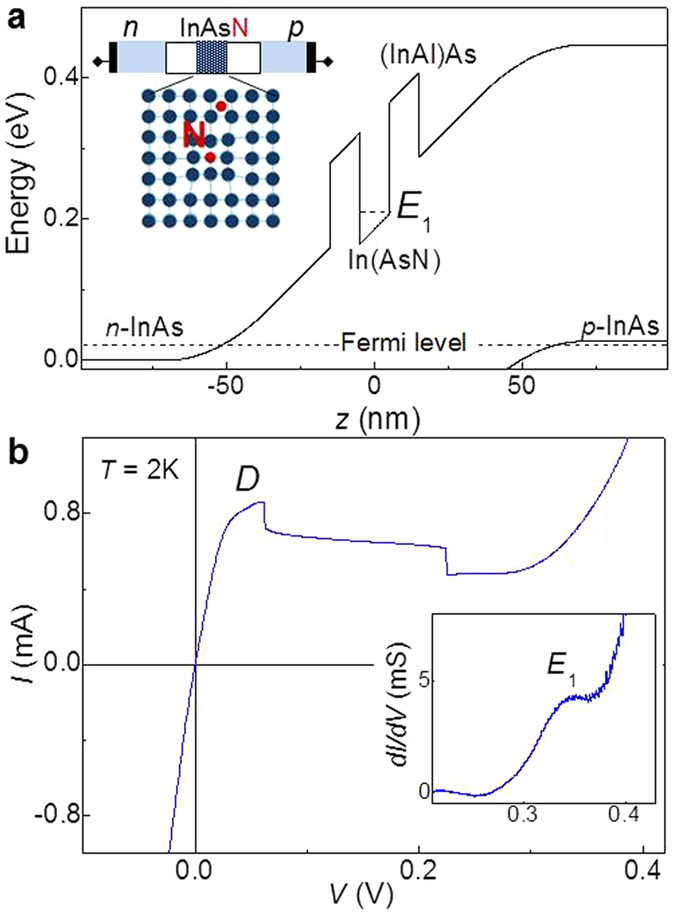
Resonant tunnelling diodes based on the narrow-gap In(AsN). (**a**) Energy band diagram of a *p-i-n* In(AsN)/(InAl)As resonant tunnelling diode (RTD) at thermal equilibrium. The black dotted lines indicate the Fermi energy and the lowest quasi-bound electron state *E*_1_ of the In(AsN) QW. Inset: Sketch of the *p-i-n* diode with the In(AsN) QW in the intrinsic (*i*) layer, and substitutional and interstitial N-atoms in InAs. (**b)** Current-voltage *I*(*V*) curve at *T* = 2 K for an In(AsN) RTD with mesa diameter *d* = 100 μm showing a strong peak *D*. Inset: d*I*(*V*)/d*V* curve showing a weak resonant feature *E*_1_.

For these studies, we designed and grew by molecular-beam epitaxy (MBE) a *p-i-n* In(AsN) RTD whose schematic band diagram at zero applied bias is shown in Fig. 1a. The i-region of the RTD consists of a 10 nm-wide In(AsN) quantum well (QW), with N-content *x* = 1%, embedded between two 10 nm (InAl)As tunnel barriers (see Methods section). Figure 1b shows the low temperature (*T* = 2K) current-voltage, *I*(*V*), characteristic for this RTD. In the following, we define positive bias with the top *p*-type layer biased positive. For low applied biases of both polarities, the *I*(*V*) curve exhibits an ohmic region; with increasing forward bias the current rises to form a broad peak *D* with a maximum at *V* ~ 0.06 V, followed by a region of negative differential resistance, NDR, in which the DC current decreases in two well-defined steps. These steps are accompanied by a self-sustained oscillatory current, which arises from the instability of the device circuit induced by the NDR. Closer inspection of the *I*(*V*) and differential conductance *dI*(*V*)*/dV* curve (see inset of Fig. 1b) also reveals a broad feature, *E*_1_, at *V *~ 0.35 V.

**Figure 2:**
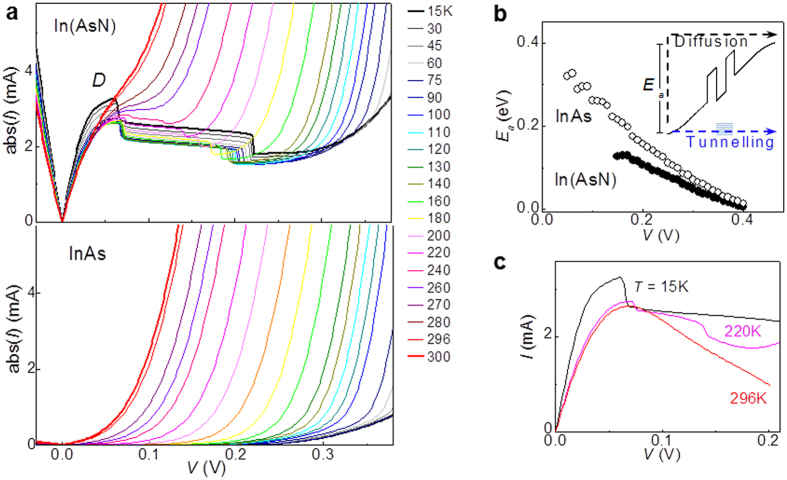
Temperature dependence of resonant Zener tunneling. (**a**) Current-voltage *I*(*V*) characteristics at temperatures *T* from 15 K to 300 K of In(AsN) (top) and InAs (bottom) RTDs with mesa diameter *d* = 200 μm. (**b**) Bias dependence of the activation energy *E*_*a*_ describing the dependence of the current *I* on temperature, *i.e. I* = *I*_*0*_exp(−*E*_*a*_/*k*_*B*_*T*). Inset: Electron diffusion and tunnelling. (**c**) *I*(*V*) characteristics of the In(AsN) RTD at *T* = 15 K, 220 K and 296 K after subtraction of the diffusion current.

The temperature dependence of the *I*(*V*)s for an In(AsN) RTD is compared with that for a control sample with no nitrogen in Fig. 2a. The striking difference between the two sets of *I*(*V*) curves is the absence of the strong resonant tunnelling peak *D* and associated NDR in the control device. This is a clear indication that peak *D* arises from tunnelling of electrons through quantum states in the In(AsN) QW layer. Also, Fig. 2a (top panel) shows that peak *D* is weakly affected by temperature over an extended temperature range up to *T* ~ 200 K. In contrast, the *I*(*V*) curves of the control sample exhibit a conventional diode-like behaviour (Fig. 2a, bottom panel).

### Resonant Zener tunnelling into N-induced zero-dimensional states

Since peak *D* occurs at biases (*V* ~ 0.06 V) that are much lower than the built-in potential (*V* ~ 0.4 V) of the diode and is absent from the control device, we exclude the possibility that it arises from tunnelling of charge carriers through the 2D subband states of the In(AsN) QW, which generates only a weak feature, *E*_*1*_, at a significantly higher bias, *V* ~ 0.35 V, close to the flat-band voltage (inset of Fig. 1b). We propose instead that *D* is due to Zener tunnelling of electrons through the forbidden energy gap between the *n*- and *p*-sides of the diode. Zener tunnelling in conventional diodes can cause a significant current when there is spatial overlap between occupied states in the conduction band of the *n*-layer and the empty states near the top of the valence band in the *p*-layer. The strength of the Zener tunnel current is enhanced for the case of a narrow energy band gap, thin barrier width and/or small carrier effective mass^2^,^16^. Since the carrier effective masses and band gap energy of InAs and In(AsN) with N = 1% are very similar (see Supplementary Material S1), one should expect a comparable weak Zener tunnelling current in both devices. The presence of the strong resonance *D* only in the In(AsN) RTD is evidence that Zener processes are enhanced by tunnelling processes through localized states in the energy gap of the In(AsN) layer.

The weak *T*-dependence of peak *D* indicates that the N-induced levels are strongly confined, thus providing an effective path for tunnelling of electrons at various *T*. We note instead that for large positive biases (*V* > 0.2 V), the *I*(*V*) curves of the InAs and In(AsN) RTDs and their temperature dependence are very similar (Fig. 2a). In this bias regime, thermal diffusion of carriers over the junction’s energy barrier represents the dominant contribution to the current and the *T*-dependence of *I* is well described by a thermally activated behaviour, *i.e*. *Ι* ~ exp(−*E*_*a*_/*k*_*B*_*T*), where *E*_*a*_ is an activation energy that depends on the applied bias (Fig. 2b): *E*_*a*_ decreases with increasing *V*, extrapolating to zero at the flat-band bias, *V* ~ 0.4 V, corresponding to the built-in potential of both diodes. Figure 2c shows representative *I*(*V*)s for the In(AsN) RTD after subtraction of the contribution of diffusion current, as measured in the device without nitrogen in the QW: peak *D* and the region of NDR are only weakly affected by temperature and can be clearly seen up to room temperature, consistent with electrons tunnelling into strongly localized 0D states.

### Magneto-tunnelling spectroscopy of zero-dimensional states

The resonant transmission of electrons through N-induced localized states is qualitatively different from Zener tunnelling of electrons in GaAs/AlAs QW *p-i-n* RTDs, where quasi-localized electric-field dependent states in the QW modulate the Zener current under an applied reverse bias^17^,^18^, and from Zener tunnelling via the gate-induced localized states of narrow-gap double-quantum-wire structures^19^. Probing the spatial form and size of the wavefunction of a 0D state require sensitive techniques, such as scanning tunnelling microscopy (STM)^20^ or magneto-tunnelling spectroscopy (MTS)^21^,^22^,^23^, which enables studies of localized states below a surface, the condition frequently encountered in semiconductor devices, such as in our RTDs.

**Figure 3:**
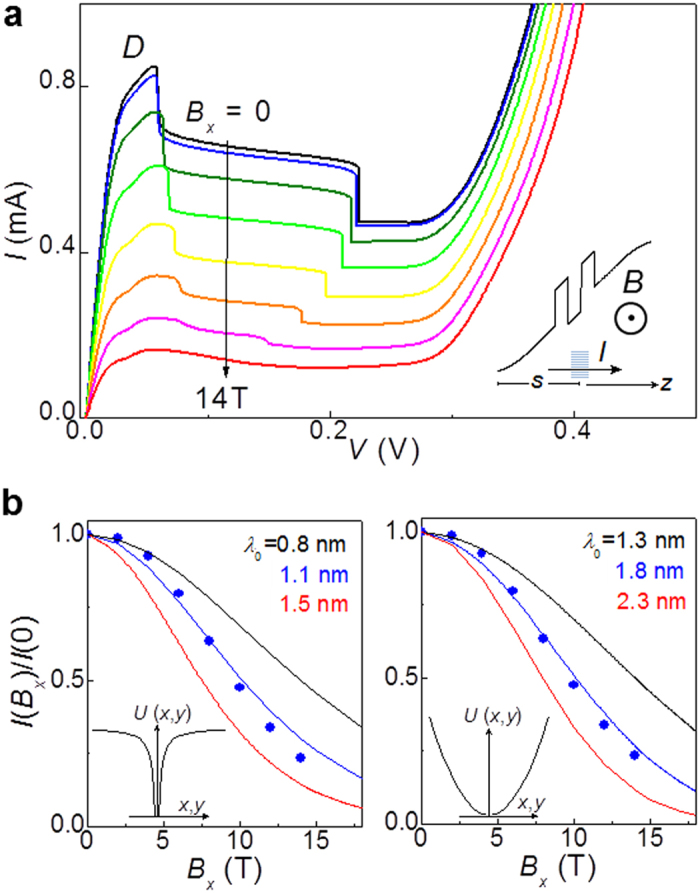
Magneto-tunnelling spectroscopy of zero dimensional states. (**a**) Current-voltage *I*(*V*) curves at magnetic fields *B*_*x*_ = 0, 2, 4, 6, 8, 10, 12, 14 T, applied perpendicular to the direction *z* of the current for an In(AsN) RTD with mesa diameter *d* = 100 μm (*T* = 2 K). (**b**) Measured (dots) and calculated (lines) dependence of the normalized current *I*(*B*_*x*_)/*I*(0). The model shown in the two panels is for a Coulomb (left) and an harmonic (right) potential *U*(*x*, *y*). In each panel, different curves correspond to different sizes λ_0_ of the electronic wavefunction.

MTS exploits the effect of the classical Lorentz force on the motion of an electron: when an electron tunnels from the emitter into the 0D state, it acquires an additional in-plane momentum given by *ħ*Δ*k*_*y *_= −*eB*_*x*_*s*, where *s* is the tunnelling distance along the tunnelling direction *z* from the emitter to the localized 0D state and *B*_*x*_ is the magnetic field applied perpendicular to *z* (inset of Fig. 3a). The variation of the tunnel current with *B*_*x*_ ∝ Δ*k*_*y*_ is determined by the size of the matrix element that governs the quantum transition of an electron as it tunnels into the 0D state and hence probes the electron probability density in Fourier space of the localized state^23^.

**Figure 4:**
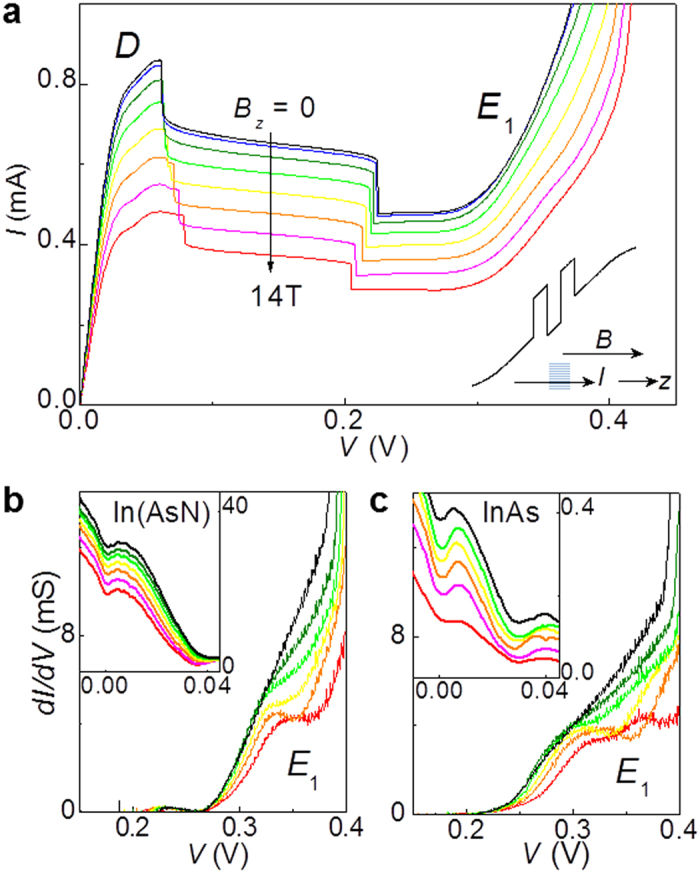
Resonant Zener tunnelling in magnetic field parallel to the direction of current. (**a**) Current-voltage *I*(*V*) curves at magnetic fields *B*_*z*_ = 0, 2, 4, 6, 8, 10, 12, 14 T, applied parallel to the direction *z* of the current for an In(AsN) RTD with mesa diameter *d* = 100 μm (*T* = 2 K). (**b,c**) Differential conductance *dI*(*V*)*/dV* curves versus *B*_*z*_ in the bias region of electron tunnelling into the first subband, *E*_1_, of the In(AsN) (**b**) and InAs (**c**) QW for *B*_*z*_ = 0, 4, 6, 8, 10, and 14 T. The insets show the *dI*(*V*)*/dV* curves at small applied biases (*T* = 2 K).

Figure 3a shows the *B*_*x*_-dependence of the *I*(*V*) curve: with increasing *B*_*x*_, the intensity of peak *D* displays a marked decrease, stronger than that observed at high bias-voltages (*V* > 0.2 V). As shown in Fig. 4a, a magnetic field applied parallel to the direction of current (*B*_*z*_) has instead only a weak effect on peak *D*. Figure 3b compares the measured *B*_*x*_-dependence of the peak current height of feature *D* with that calculated using a transfer Hamiltonian model of resonant tunnelling through a localized state for two different forms of the confining potential (insets of Fig. 3b). In our model, we assume separability of the 0D wavefunction into in-plane (*xy*) and normal (*z*) components, and express the normalized tunnel current, *I*(*B*_*x*_)/*I*(0), as │*M*(*B*_*x*_)/*M*(0)│^2^, where *M*(*B*_*x*_) and *M*(0) are the overlap integrals in the *xy*-plane of the emitter and the N-induced localized states for *B*_*x*_ > 0 and *B*_*x*_ = 0, respectively. The calculated and measured magnetic field dependencies are discussed in the following section and differ from those expected for Zener tunnelling between extended states in magnetic field^24^.

## Discussion

**Figure 5:**
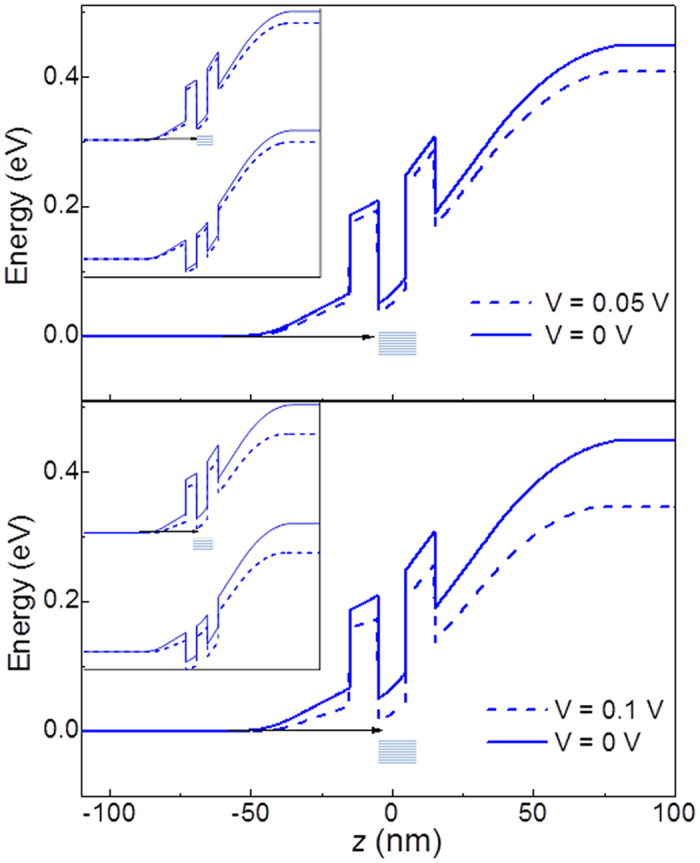
Resonant Zener tunnelling via zero dimensional states. Calculated profile of the conduction band edge at applied biases *V* = 0 V (continuous line) and *V* = 0.05 V, 0.1 V (dashed lines). The arrow sketches tunnelling of electrons from the *n*-emitter layer into localized states of the In(AsN) layer (shaded rectangle). Insets: Profile of both the conduction and valence band edges under the same bias conditions illustrated in the main panels.

We model the energy band diagram of the RTD by solving Schrödinger and Poisson equations, and by taking into account changes due to nitrogen of fundamental band parameters, *e.g.* the band gap energy and electron effective mass of InAs (see Supplementary Material S1). The N-incorporation in the InAs layer leads to a lowering of the energy of the quasi-2D ground state subband of the QW by 30 meV compared to InAs. Also, nitrogen introduces donor states with density *n*_D_ ~ 3 × 10^17 ^cm^−3^ for *x* ~ 1%, as measured from Hall studies of In(AsN) epilayers^25^. Figure 5 shows the CB potential profile of the RTD at thermal equilibrium for a concentration of ionized donors *n*_D_ = 3 × 10^17 ^cm^−3^ in the QW, which create a non-uniform electric field compared to *n*_D_ = 0. When a small voltage, *V*, is applied (Fig. 5, top panel), resonant tunnelling through a particular N-related energy state in the gap of In(AsN) should lead to a resonance in *I*(*V*), whenever a filled state in the negatively biased electron emitter *n*-layer is resonant with an adjacent empty localized N-related state in the In(AsN) layer. Since these states may be located at different positions in the well, we can expect a distribution of energy levels and/or tunnelling distances, and correspondingly a broadened resonance. For sufficiently high bias voltages, no states are available for electron tunnelling (Fig. 5, bottom panel) and the current decreases, thus leading to NDR in *I*(*V*).

From the band profile in Fig. 5 and the measured *I*(*V*) curve, we estimate that the 0D states lie at an energy *E*_0_ > 40 meV below the CB minimum of In(AsN). This is significantly larger than the binding energy for a shallow donor in InAs (*E*_0_ ≈ 1.6 meV) and is consistent with the weak dependence on temperature of peak *D.* Using the evanescent wave model of a defect state in the energy gap, we estimate the spatial extent of the defect. For a state at an energy of *E*_0_ below the CB minimum, the electron wave function in the *xy*-plane is *ψ*(*x*, *y*)~*e*^−*ρκ*^ with 
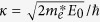
 and *ρ *= (*x*^2 ^+ *y*^2^)^1/2^. Using 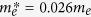 and *E*_0_ > 40 meV, we derive a typical length κ^−1^ < 5.9 nm, much smaller than for a shallow donor in InAs.

We can draw similar conclusions by considering the *B*_*x*_-dependence of the peak *D* (Fig. 3). To describe this dependence, we consider different forms for the confinement potential. For a Coulomb potential of the form *U*(*x*, *y*) = −*e*^2^/(4*πγε*_0_*ε*_*r*_*ρ*), where *ε*_*r*_ is the relative permittivity, *ρ *= (*x*^2 ^+ *y*^2^)^1/2^, and *γ* is an empirical parameter describing the effect of the QW potential on the donor, the electron wave function in the *xy*-plane is described by 
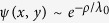
, where *λ*_0_ is an effective Bohr radius^26^. However, this long-range potential may not be valid to describe the localized states arising from N-related defects. In particular, a short-range confining potential can also be induced in the QW by a small number of closely spaced electrical charges associated to donors and/or acceptors^27^, or a variation in the N-composition. Since such a potential should be approximately parabolic close to its minimum, its ground state can be approximated by a simple harmonic wavefunction. Thus for a parabolic potential 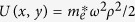, where 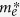 is the electron effective mass and *ω* is the angular frequency of the harmonic oscillator, the wavefunction in the *xy*-plane is given by 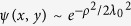, where 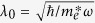.

For the Coulomb and parabolic potentials, respectively, we express the *B*_*x*_-dependence of the current as^23^,^26^:


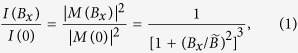


and


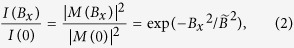


where 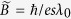, and *s* is the average tunnel length from the emitter to the bound state. From the calculated band energy profiles, we estimate *s* ~ 30 nm for *V* = 0.05 V. As shown in Fig. 3b, the calculated *I*(*B*_*x*_) curves reproduce accurately the data if we take λ_0_ = (1.1 ± 0.1) nm for the Coulomb potential and λ_0_ = (1.8 ± 0.1) nm for the harmonic potential. Although we cannot establish the specific form of the wavefunction, we can conclude that the N-induced states responsible for peak *D* are strongly confined. Using a 3D (2D) hydrogenic model, the value of λ_0_ gives an *effective* Rydberg energy *E*_0_ = 30 meV (120 meV). Our estimate of the spatial extent of the defect is consistent with the evanescent wave model of a defect state in the energy gap.

A magnetic field applied parallel to the direction of current (*B*_*z*_) has a weaker effect on Zener tunnelling, see Fig. 4a. For this orientation of *B* up to 14 T, the magnetic confinement length in the plane of the QW, *l*_*z *_= (*ħ*/*eB*_*z*_)^1/2^, is always larger than λ_0_: for *B*_*z*_ = 14 T, *l*_*z*_ = 6.8 nm > λ_0_. Thus the N-localized states are not greatly modified by the confining potential of the magnetic field; also, they have a weak effect on the tunnelling of electrons into the QW subbands, see feature *E*_1_ in the *dI*(*V*)/d*V* curve in Fig. 4b. Tunnelling of electrons into the lowest Landau level (LL) of the first QW subband (*n* = 1) produces a small diamagnetic shift of *E*_1_ with increasing *B*_*z*_, as is also observed in the control sample (Fig. 4c). A modulation of the d*I*(*V*)/d*V* curve due to tunnelling of electrons into the first two LLs can also be seen in the control sample, where *E*_1_ splits into distinct features at *B*_*z*_ > 10 T. Closer inspection of the *dI/dV* curves reveals a series of weak, but sharp resonant features at *V* = 0 and 0.03 V (inset of Fig. 4b,c). These are observed in all RTDs (with and without nitrogen) and are assigned to tunnelling of electrons from the *n*- to the *p*-sides of the diode without and with emission of longitudinal optical (LO) phonons, which have energy *ħω*_LO_ = 29 meV.

The effects of the incorporation of N on the electronic band structure of several III-Vs has been examined extensively in earlier work^11^,^12^,^13^,^14^,^15^. The majority of these studies has focused on wide band gap (InGa)(AsN) compounds, where the incorporation of N leads not only to isolated substitutional N-atoms but also to the formation of N-N pairs, where a group III atom has two N nearest neighbors, and of higher-order N clusters with energies below and above the conduction band minimum. In the narrow band gap alloy In(AsN), the same clusters are expected to lie well-above the conduction band edge^13^ and thus they cannot be responsible for the localized energy levels in the band gap that we have identified in our tunnelling experiments. On the other hand, non-substitutional N-related crystal defects are known to form in dilute nitrides^14^,^15^,^28^, including In(AsN)^29^. For our In(AsN) layers, we have conducted high-resolution x-ray diffraction (HRXRD) studies to evaluate the N-composition and crystal quality. The HRXRD studies were then compared with time-of-flight secondary ion mass spectroscopy (ToF-SIMS) to assess the purity of the layers and the total amount of N^29^. The N-content obtained by ToF-SIMS was found to be higher (by about 5%) than that derived from the analysis by Vegard’s law of the HRXRD data and that the difference increases with increasing N-content^29^. This behaviour, which was also observed in the dilute nitride Ga(AsN) alloy^28^, suggests that non-substitutional N atoms are incorporated into the crystal lattice. Non-substitutional configuration of N may include N–As split interstitials, N-antisites, and/or interstitial-N. The identification of the specific crystal defect and the modelling of its energy levels require further investigations.

In summary, we have presented the first experimental study of a RTD based on the MIR alloy In(AsN). The *I*(*V*) characteristics at low *T* show an extended region of NDR as well as an ohmic behaviour around zero bias that we attribute to Zener tunnelling of electrons from the *n-* to the *p*-side of the diode, assisted by N-induced levels. The weak temperature dependence of the NDR over an extended temperature range indicates that the levels are confined in the In(AsN) QW layer. Magneto-tunnelling spectroscopy demonstrates that these levels are localized over a characteristic length λ_0_ ~ 1.5 nm and lie below the conduction band minimum of In(AsN). The presence of N-related defects is known to be the main cause of deterioration of both optical and electrical properties of wide band gap dilute nitrides. For the narrow band gap In(AsN) alloy, although disorder effects tend to be significantly weaker due to the different energy position of the N-N pairs and N-clusters, the incorporation of nitrogen can be still accompanied by the creation of localized states in the energy gap. Tunnelling of carriers into these states could increase leakage currents and noise levels in optical and electrical devices, such as MIR LEDs and photodetectors. Our RTD structures allow us to sensitively probe such states; also, electron tunnelling through these states give rise to NDR that is only weakly affected by temperature, thus offering potential applications for non-linear components in electronics and MIR photonics.

## Methods

### Growth and fabrication

The In(AsN) and InAs RTDs were grown by MBE. The In(AsN) RTD is a multilayer structure consisting of a 10 nm-wide In(AsN) quantum well (QW), with N-content *x* = 1%, embedded between two 10 nm (InAl)As tunnel barriers with Al-content *y* = 10%. Undoped InAs spacer layers of width 20 nm separate the (InAl)As barriers from 10^17 ^cm^−3^ *n*-doped and *p*-doped InAs layers of width 300 nm on each side of the barriers. The above layers were sandwiched between 10^18 ^cm^−3^ *n*- and *p*-doped InAs layers of width 100 nm, which formed low resistance electrical contacts. The structure was grown on an *n*^+^-type InAs substrate at 500 °C except for the In(AsN) layer, which was grown at 380 °C. For comparison, we also studied a control sample with the same sequence of layers as that described above, except that it contains no nitrogen and the InAs QW layer is grown at 460 °C. These growth temperatures were chosen to optimize the quality of the layers, as assessed by preliminary structural, Raman and photoluminescence studies of In(AsN) layers with different N-concentrations. The samples were processed into circular mesa structures of diameter *d* = 100 and 200 μm, with ohmic contacts alloyed to the top and bottom doped InAs layers.

### Transport measurements

The measurements of the *dc* dark current versus the applied voltage were made using a Keithley2400 source-meter. The magneto-transport studies were performed in a superconducting magnet generating magnetic fields up to 14 T.

## Additional Information

**How to cite this article**: Di Paola, D. M. *et al*. Resonant Zener tunnelling via zero-dimensional states in a narrow gap diode. *Sci. Rep.* **6**, 32039; doi: 10.1038/srep32039 (2016).

## Supplementary Material

Supplementary Information
