# Supplementary material for: Resonant Zener tunnelling via zero-dimensional states in a narrow gap diode
Source: Sci Rep. 2016 Aug 18;6:32039. doi: 10.1038/srep32039 (PMC4989182; doi:10.1038/srep32039)
Supplement: Supplementary Information [file srep32039-s1.pdf]

Supplementary Material

"Resonant Zener tunnelling via zero-dimensional states in a narrow gap diode"

D.M. Di Paola[1], M. Kesaria[2], O. Makarovsky[1], A. Velichko[1], L. Eaves[1], N. Mori[3], A. Krier[2], A. Patanè[1]*

[1]*School of Physics and Astronomy, The University of Nottingham, Nottingham NG7 2RD, UK*

[2]*Physics Department, Lancaster University, Lancaster LA1 4YB, UK*

[3]*Graduate School of Engineering, Osaka University, 2-1 Yamada-Oka, Suita City, Osaka 565-0871, Japan*

We model the energy band diagram of the InAs/(InAl)As and In(AsN)/(InAl)As quantum wells (QWs) by solving the Schrödinger equation in the effective-mass approximation with the band parameters from Tables 1 and 2. The InAs and In(AsN) layers have width w = 10 nm and are embedded between two 10 nm (InAl)As barriers with Al-content of 10%. For In(AsN), the nitrogen-content is N = 1%.

	InAs	In(AsN), N = 1%	(InAl)As, Al = 10%
m_e^*	$0.025m_0$	$0.026m_0$	$0.039m_0$
E_g at T = 4.2K (eV)	0.415	0.378	0.59

Table 1: Electron effective masses (m_e^*) and band gap energies (E_g) for bulk InAs, In(AsN), and (InAl)As. For In(AsN), we take into account the reduction of the conduction band (CB) edge due to N using the band anticrossing model [1]. We assume that the N-level, E_N, is at 1.44 eV above the valence maximum and that the coupling between the N-level and the CB states is V_N =2.0 eV. As found in ref. [2], the electron effective mass is only weakly affected by nitrogen for N = 1%.

	InAs/(InAl)As	In(AsN)/(InAl)As, N = 1%
eV_e (eV)	0.12	0.16
E_1-E_C (eV)	0.045	0.053

Table 2: Height, eV_e, of the InAs/(InAl)As and In(AsN)/(InAl)As quantum wells (QWs) and energy E_1 of the first QW resonant state. E_1 is calculated relative to the CB minimum, E_C, of InAs and In(AsN). For the InAs/(InAl)As QW, we use for eV_e the same value as in reference [3]. For In(AsN), the value of eV_e is increased due to the N-induced red-shift of the CB minimum.

Supplementary Material

Figure S1 shows the profile of the CB minimum and the energy of the first QW resonant state E_1 for each QW. The effect of the N-incorporation is to lower the energy of the CB minimum and the QW resonant state E_1 by ~ 40 meV and 30 meV, respectively. Tunnelling of electrons through the 2D subband states of the In(AsN) QW generates a weak feature, E_1, at a bias, $V \sim 0.35$ V, which is similar to that observed in InAs (Fig. 4b and 4c in the manuscript). Peak D is peaked at much lower biases ($V < 0.06$ V) than the E_1 resonance, thus suggesting that D arises from tunnelling of electrons onto N-induced zero-dimensional states below the QW subband.

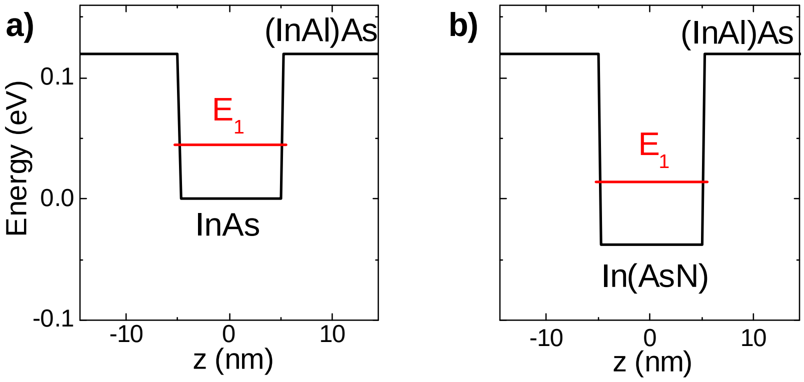

Figure S1. Profile of the conduction band minimum for InAs/In(AlAs) (a) and In(AsN)/In(AlAs) (b) quantum wells (QWs). The red line shows the energy position of the first QW resonant state E_1.

References

1 O'Reilly, E.P., et al., Trends in the electronic structure of dilute nitride alloys. *Semicond. Sci. Technol.* **24** (033001) (2009).

2 Drachenko, O., et al., Cyclotron resonance mass and Fermi energy pinning in the In(AsN) alloy. *Appl. Phys. Lett.* **98** (162109) (2011).

3 Lin, H.-K., et al., Design and characteristics of strained InAs/InAlAs composite-channel heterostructure field-effect transistors. *J. Appl. Phys.* **97** (024505) (2005).
